# Epidemiology of middle ear and mastoid cholesteatomas. Study of 1146 cases

**DOI:** 10.1590/S1808-86942011000300012

**Published:** 2015-10-19

**Authors:** Jose Evandro Andrade Prudente de Aquino, Nelson Alvares Cruz Filho, Julia Negro Prudente de Aquino

**Affiliations:** 1 Assistant professor; 2 Assistant professor of otorhinolaryngology. In charge of the Otology Unit, Beneficência Portuguesa Hospital, SP; 3 Third year resident in otorhinolaryngology, Cema, SP. Paulista Medical School (EPM), Unifesp. Otology Outpatient Unit - Prof. Dr. Nelson Alvares Cruz

**Keywords:** cholesteatoma, epidemiology, ear, middle

## Abstract

Middle ear cholesteatoma is an important and relatively common disorder which may have serious consequences.

**Aim:**

The purpose was to conduct a retrospective study of the statistics of 1,146 middle ear surgical procedures for middle ear cholesteatoma in adults and children of low income living in distant areas from our city.

**Methods:**

From 1962 to 1988 there were 1,146 surgeries for unilateral or bilateral cholesteatomas in children and adults, which were reviewed for the following criteria: total number of surgeries, sex, onset of the first symptoms, duration of the disease, the site of perforation, the cholesteatoma site, changes in the ossicular chain, the contralateral ear, bilateral cholesteatomas, the site of residual cholesteatoma, and complications.

**Results:**

Results are shown graphically on a pie chart.

**Conclusion:**

The etiology of cholesteatomas remains unknown. Epidemiological and statistical data, surgical reports, and conclusions of experimental studies are welcome, as they may provide support for clarifying the pathogenesis of cholesteatoma. Our results were compared with internationally published papers. We found no published papers on the epidemiology of cholesteatoma in the Brazilian literature.

## INTRODUCTION/OBJECTIVE

The middle ear cholesteatoma is one of the most fascinating and complex topics in otology when studying its various aspects in depth. This topic has stimulated much research and debates worldwide.

It is an important and relatively common disease that may have serious consequences. Sade et al.^1^ have shown that 0.5% to 30% of any given community has chronic otitis media; it is estimated that over 20 million people worldwide are affected. Of these, one fourth (about 5 million) have cholesteatomas. The severity of this disease is a consequence of its effects, namely compression and infection. With time, it destroys the ear, resulting in hearing loss or deafness in most patients with this condition. At times, there may be a risk against their life.

This disease has been studied since ancient times; it was widely treated, although its detailed causes were poorly known. The name is controversial. The literal transcription comes from two Greek words: *chole* (bile) and *steatoma* (fatty tumor). Its location in the middle ear differentiates it from crystalline pearl tumors that are generally found in the endocranium. In its preferred site (the middle ear or the temporal bone), cholesteatomas are epidermoid structures with corneal degeneration.

The main factors that contribute to the development and frequency of cholesteatomas are: geography, genetics, sex, age, the environment, the social and economic status, health, incorrect use of antibiotics, and others.

Statistical epidemiologic studies may be used, but they are rather limited. There are reports that one or two Eskimos have chronic otitis media, but that the incidence of cholesteatoma is very low. Among the Canadian population, its incidence is high in whites and low in blacks. It appears that genetic, anatomic, and physiological changes, added to geographical or environmental factors, affect the incidence of this disease. There appears to be no significant gender difference. Except for the congenital cholesteatoma, the incidence of this disease is higher in the elderly.

The incidence of cholesteatomas in childhood and adolescence appears to depend on the environment, on social and economic factors, and on health.^1^ It is related with secretory otitis in infancy, from before the advent of antibiotics, although this appears to have been neglected for a while. The level of healthcare in different countries appears also to affect the incidence of this condition.^1^

Pediatricians and general practitioners often treat acute otitis, secretory otitis, and rhinopharyngeal diseases; these professionals at times do not evaluate these cases as required, and may prescribe antibiotics indiscriminately. It is well known that these conditions may foster the development of cholesteatoma.

This disease, as well as its subsequent effects on hearing, may become less frequent worldwide as pediatric otorhinolaryngology develops.^1^

Except for histological studies of temporal bones, the epidemiology of cholesteatomas has been poorly studied in Brazil. As it is not a disease that has to be notified, the true incidence of middle ear cholesteatoma is unknown in this country.

The purpose of this study was to carry out a retrospective evaluation of the statistics of 1,146 surgeries for middle ear cholesteatoma in adults and children from all regions in Brazil.

## REVIEW OF THE LITERATURE

The incidence of cholesteatoma varies worldwide, depending on each population. Bezold^2^ has suggested that auditory canal dysfunction causes retraction of the tympanic membrane in cholesteatomas; the author recommended adenoidectomy as a preventive approach. Nager^3^ reviewed 12,000 patients with chronic middle ear discharge and found cholesteatomas in one third of cases. Tumarkin^4^ and Jain^5^ have suggested that economic factors may influence the pathogenesis of chronic otitis media, with reflexes on the epidemiology of cholesteatomas. Sade et al.^6^ studied the prevalence of cholesteatomas among ethnic groups at a hospital in Israel, showing that the ethnic distribution of bilateral perforation because of cholesteatomas differed significantly compared to other ear diseases. This difference is due to geogenes, rather than genes that have already been found. The annual rate of surgery for cholesteatoma has been estimated at 66 per 100,000 inhabitants/year. Schuknecht^7^ has suggested that the epithelial pouch of cholesteatomas is dry; thus, keratin may accumulate slowly for years without causing complications; if an infection occurs, cholesteatomas may develop rapidly. Although not contributing to the pathogenesis of cholesteatomas or otitis media in climatic conditions, it may increase the frequency and severity of infection, accelerating the growth of a cholesteatoma and increasing disease severity. Harker^8^ reported an annual incidence of 6 cholesteatomas per 100,000 inhabitants/year in a population in Iowa, US; the incidence peaked in the second and third decades of life. Hatnesar^9^ has suggested that the prevalence is lower in Eskimos, suggesting that their anatomic and morphological features could facilitate aeration of the middle ear and thereby avoid the complications of chronic otites.

In the US, Ruben^10^ has shown that the incidence of cholesteatomas is 4.2 cases per 100,000 inhabitants/year from 18 new chronic otitis media cases with or without cholesteatomas. Tos^11^ found an annual incidence of cholesteatomas of about 3 children and 12.6 adults per 100,000 inhabitants/year in a study of 137 cholesteatomas in children and 603 cholesteatomas in adults, operated during a 16 year period. Van Cauwenberge et al.^12^ studied 54 patients to investigate their clinical history of ear diseases; acute recurring otitis media was the most important disease. Middle ear effusions may predispose to chronic otitis media, of which cholesteatomas are a part. In the present study, the prevalence of cholesteatomas following tympanectomy was lower than 0.5%.

Manolidis et al.^13^ studied the epidemiology of cholesteatomas in Greece from 1960 to 1987 and found an equal frequency among patients of all social classes. Padgham et al.^14^ found an annual incidence of 13 cholesteatomas per 100,000 inhabitants/year from 1966 to 1986 in Scotland. Homoe & Bretlau^15^ found cholesteatomas in 35 Greenlandic Inuit in 756 operations for chronic otitis media in Greenland from 1976 to 1991. The incidence was calculated at 5 cases per 100,000 inhabitants/year, about two new cases of cholesteatoma/year. Kempainen et al.^16^ and Chinski^17^ have stated that cholesteatomas has a similar incidence among social groups; these authors showed that grommets placed in the ear reduce the prevalence of this disease. Nelson et al.^18^ divulged the incidence of cholesteatomas as being about 1.4 time higher in men compared to women. These authors reported that the mean age of children with congenital cholesteatoma was 5.6 years; the mean age in children with the acquired disease was 9.7 years. Potsic et al.^19^ showed a high prevalence in Caucasian populations, followed by Afro-descendants in their epidemiological studies. Cholesteatomas were seen rarely in Asians. Olszenska et al.^20^ showed that the annual incidence of cholesteatomas was about 3 per 100,000 inhabitants/year in children, and about 9.2 per 100,000 Caucasian adults/year, with a predominance in males. Dornelles et al.^21^ has monitored 450 Brazilian patients since August 2000, and has found a 30% rate of cholesteatomatous chronic otitis media, which presented bilaterally in 12% of the sample. Of these patients, 45% were aged not more than 18 years, therefore included in the pediatric and adolescent population; male patients comprised 70% of the cases.

## METHODS

A longitudinal cohort retrospective survey was done of a statistical study of 1,146 cholesteatoma surgeries in adults and children from 1962 to 1988. For this study we selected cholesteatoma cases among about 5,000 files of patients treated medically and surgically for middle ear conditions. The series comprised 1,146 cases of cholesteatoma surgeries in adults, adolescents, and children from all regions of Brazil.

The follow-up files of all patients were complete, containing the medical history, otoscopic and microscopic examination of the ear, nose, throat, and the pharyngeal ostium of the auditory tube, cultures and antibiograms of ear secretions, audiology and radiologic studies.

Epidemiologic data for several aspects of cholesteatomas were studies, as follows:

1) number of procedures
2) sex
3) age of onset of the first symptom
4) the first symptom of the disease
5) duration of disease
6) perforation site
7) location
8) changes in the ossicular chain
9) the contralateral ear
10) bilateral cholesteatomas
11) the site of residual cholesteatomas.

## RESULTS

The results of parameters are shown as graphs and pie charts.^22^ (Chart 1, Chart 2, Chart 3, Chart 4, Chart 5, Chart 6, Chart 7, Chart 8, Chart 9, Chart 10, Chart 11)

**Chart 1:**
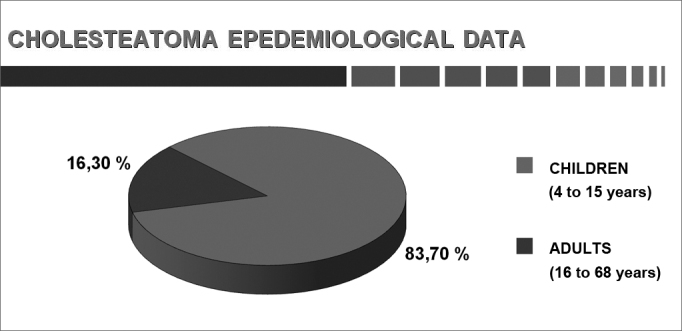
Total number of procedures.

**Chart 2:**
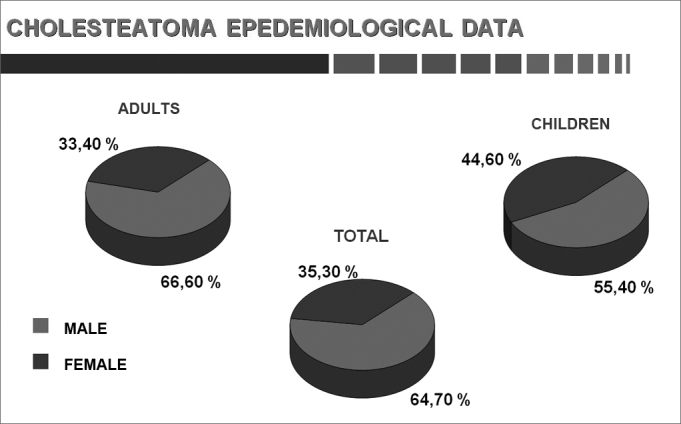
Sex.

**Chart 3:**
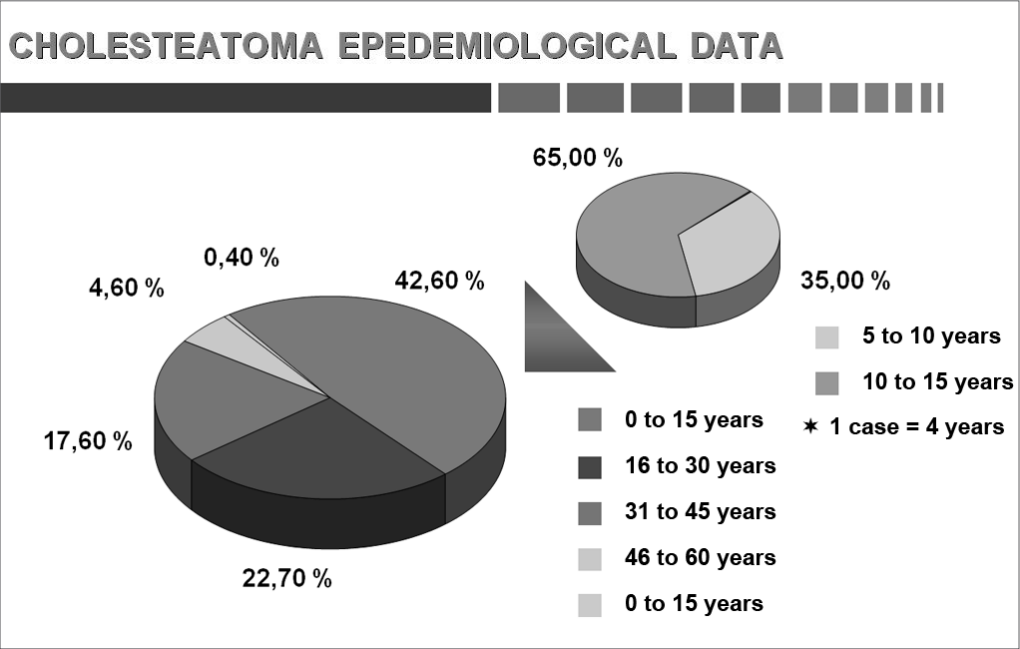
Age.

**Chart 4:**
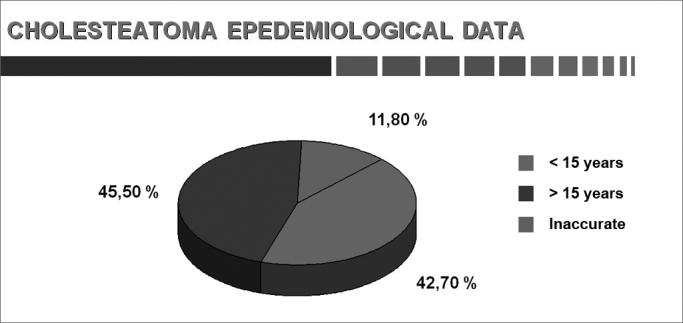
Age at onset of the first symptom (adults and children).

**Chart 5:**
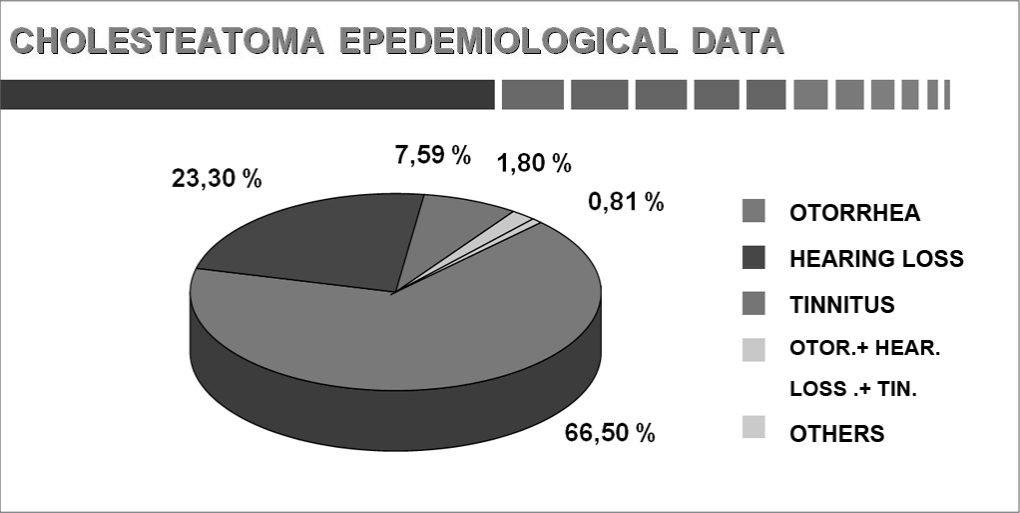
The first symptoms (adults and children).

**Chart 6:**
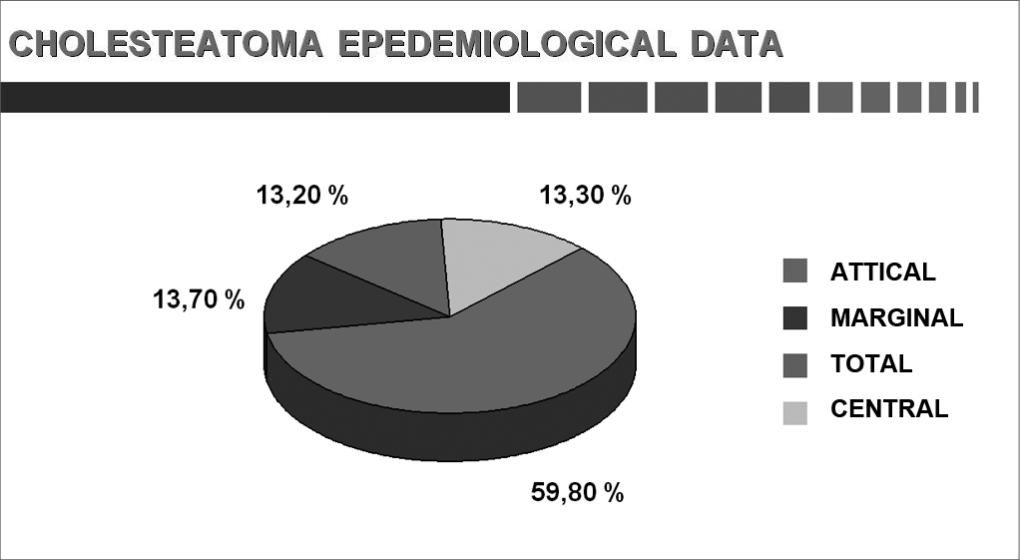
Perforation site (adults and children).

**Chart 7:**
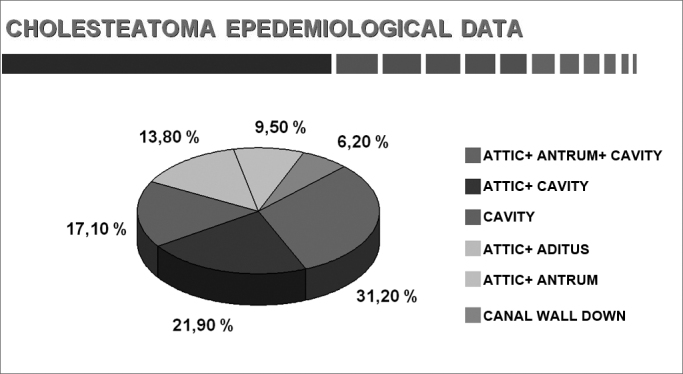
Changes in the ossicular chain (adults and children).

**Chart 8:**
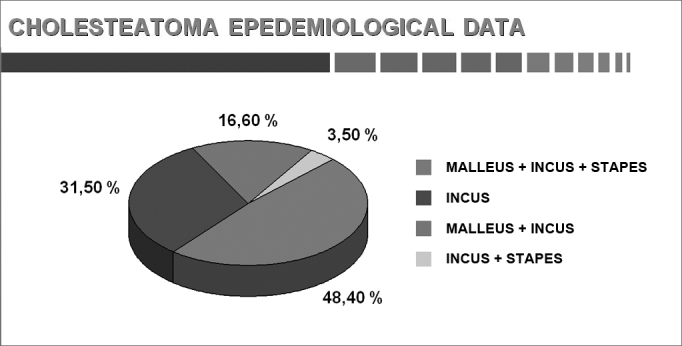
The contralateral ear (adults and children).

**Chart 9:**
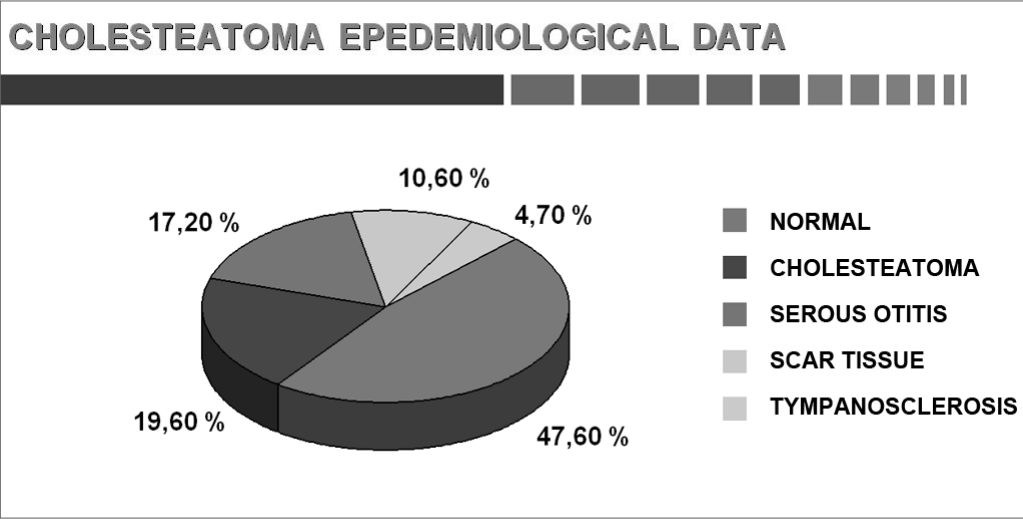
Percentage of bilateral cholesteatomas (adults and children).

**Chart 10:**
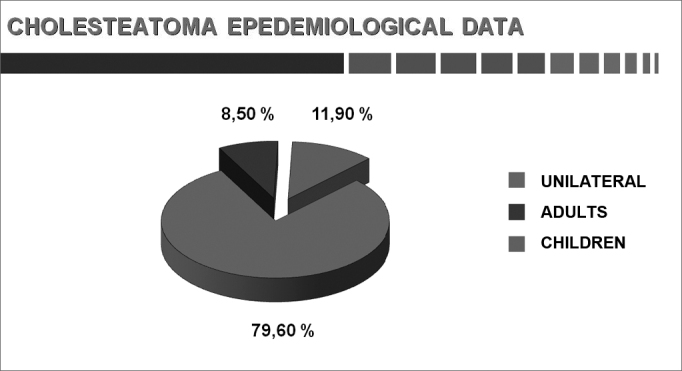
Site of residual cholesteatomas in the open technique (adults and children).

**Chart 11:**
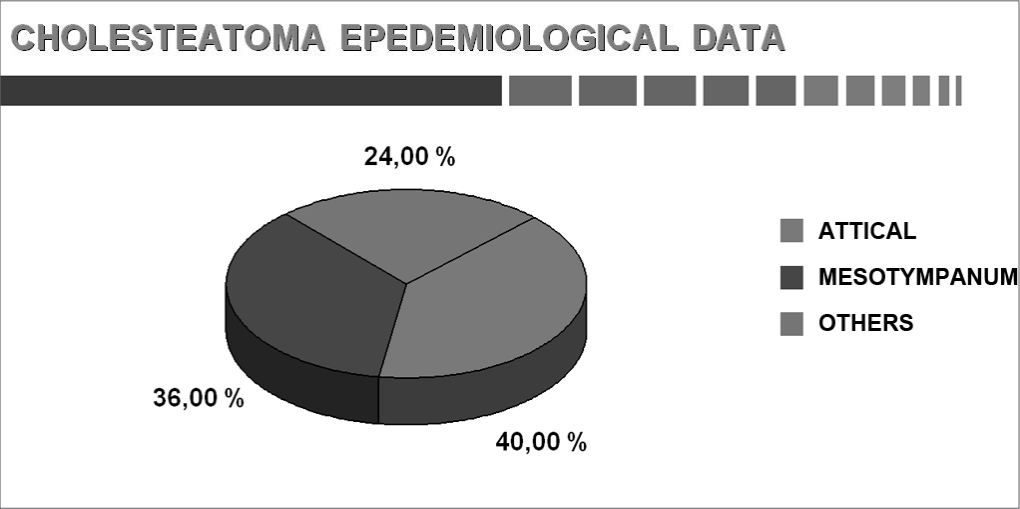
Location (adults and children).

## DISCUSSION

This study comprised 1,146 cases, of which 960 were adults and 186 were children. Each group was considered in separate. There were 639 male (66.6%) and 321 female (33.4%) adult subjects. The age of adults ranged from 16 to 68 years; the age of children ranged from 4 to 15 years. Patients aged 16 complete years were considered as adults.

Males predominated slightly (64.7%) compared to females (35.3%) among both adults and children. Sadé et al.^1^ found a male predominance (55.7%) over females (44.3%) for this disease.

The age at which patients present when cholesteatomas are diagnosed was controversial because of the low social and economic status of patients in our series; the disease was found in 45.5% of our adult patients. We calculated that the onset of symptoms in about half of patients took place before 15 years of age, suggesting that childhood is important in this disease.

The duration of the disease from the onset of symptoms varies significantly among patients. About 30% of these patients wait from 6 to 15 years before seeking medical help; they carry the symptoms for years without a diagnosis.

We believe that two important factors cause such delays in the diagnosis:

a) patients often attribute little importance to otological symptoms except when there is pain, dizziness, or bleeding;
b) cholesteatomas may be well known to ENT specialists, but is less familiar to pediatricians and general practitioners.

The first and most frequent symptom of cholesteatomas in our sample was otorrhea (66.5%), followed by combined otorrhea-hypoacusis-tinnitus (23.3%), and hypoacusis (7.6%).

Sadé et al.^1^ found that a discharge was the first symptom in 62.0% of cases; hypoacusis was present in 11.0% of cases.

We know that the perforation site on the tympanic membrane is rarely central; this occurred in 13.3% of our sample. Perforation was marginal and attical in 73.6% of cases. Sadé et al.^1^ found marginal and attical perforation in 84.0% of their cases.

Cholesteatoma may be located in any of its possible sites, although the attic was involved more frequently.

The ossicular chain is the first structure to be damaged. Two points should be made: we have never seen a damaged stirrup alone; and the most commonly damaged ossicle is the anvil. Palomar et al.^23^ has shown that the anvil was involved in 100% of ears where the ossicular chain was damaged.

The presence of middle ear cholesteatomas in patients with chronic otitis media results in a higher morbidity and mortality because of the erosion power of these epithelial growths (Sadé & Halevy^24^). Cholesteatomas usually affect the ossicular chain; less frequently, they may also involve cranial bones, including the hardest bone of the body, the optic capsule, which demonstrates its destructive power over bone. Partial or complete destruction of ossicles may be seen in 80% of patients with cholesteatomas. The ossicular chain erosion rate in chronic non-cholesteatomatous otitis media is about 20% (Chole^25^). The mechanisms by which bone is degraded in the presence of cholesteatomas remain unclear.

Swartz^26^ has suggested that destruction of ossicles is the most common complication of cholesteatomas; the type of destruction depends on the original site and expansion of the cholesteatoma. These authors found that the ossicular chain was intact in only 26% of attical cholesteatomas; the long process of the anvil was the most affected area, followed by the body of the anvil and the head of the hammer. Cholesteatomas in the portion under tension have a 90% erosion rate.

Several factors stimulate bone resorption, such as inflammation, local pressure, and specific cytokeratins (Olszewska et al.^20^). Abramson & Huang^27^ proposed the enzyme concept, in which epithelial enzymes are thought to cause bone destruction; these authors found collagenases and hydrolases in cholesteatomas. Thompsen^28^ and Ferlito et al.^29^ later confirmed this hypothesis by suggesting that collagenases produced by the components of fibrous and squamous epithelial tissues caused bone erosion. Other agents have since been added to the hypothesis of biochemical bone resorption by collagenolytic enzymes only; these include the tumor necrosis factor (TNF), interleukins (IL-1a), and prostaglandins (PGE2) (Hansen et al.^30^).

Bone resorption mechanisms in chronic otitis media are not yet fully understood. Ruedi^31^ and Tumarkin^32^ have suggested that bone resorption could occur as a result of pressure by cholesteatomas over ossicular surfaces. Thompsen et al.^33^ and Sadé & Berco^34^ found that inflammation invariably surrounded eroded ossicles, and suggested that inflammation could be the cause of ossicle resorption. Granulation tissue next to ossicles may produce several enzymes and mediators that may accelerate ossicle resorption; these include lysosomal enzymes, collagenases, and prostaglandins. The main cell that dominates the bone resorption process still is controversial. These authors have suggested that persisting inflammation in cholesteatomatous chronic otitis media could give rise to a perpetual scarring process in the perimatrix of cholesteatomas, thereby raising the level of cytokines. These, among other factors, could be accounted for the growth of cholesteatoma and the resulting bone destruction (Milewski^35^).

Ossicle alterations are part of the concept of chronic otitis media itself - irreversible inflammatory damage of tissues. The clinical effect is clear, as the conduction of sound to the inner ear is affected, resulting in conduction dysacusis of variable intensity. It is thought that chronic inflammatory changes in the ossicular chain follow a continuous and repetitive pattern. This means that the structure of ossicle is maintained in a fragile balance between its resistance and destructive mechanisms due to chronic inflammation.

The anvil was the most affected ossicle, followed by the hammer and the anvil. This is probably due to the incudal mass of the anvil, its prominent medulla, and especially the exposure and frailty of the long apophysis and its lenticular process. These factors acting synergistically appear to make this ossicle more vulnerable to extrinsic damage and to osteomyelitis. These findings have confirmed those of Tos^36^ in a review of ossicle disease in 1,150 ears with chronic otitis media; the anvil, stirrup, and hammer were shown to be more frequently affected by inflammation in that order.

It is currently thought that ossicle defects result from active bone resorption processes, rather than necrosis. This theory assumes that live cells participate in the mechanisms of bone demineralization, erosion, and destruction (Kranc et al.^37^). A necrotic bone may remain in place for years without being resorbed. This has been well illustrated in reconstructions of ossicle chains with homologous bone implants; in such cases, the ossicles remain intact in the long term, making it possible for sound to propagate through the middle ear.

Abramson^38^ and Deguine & Deguine^39^ have shown that the percentage of affected contralateral ears is about 50% in cholesteatoma cases.

Deguine & Deguine^39^ found normal contralateral tympani in one third of their cases only; cholesteatomas were present in 10% of contralateral ears. Aquino^40^ found bilateral cholesteatomas in 19.6% of cases; the contralateral ear was normal in 47.6% of cases.

Abramson^38^ and Deguine & Deguine^39^ have found that cholesteatomas are bilateral in children in more than 10% of cases; for Sheehy et al.,^41^ this rate is 8.0%.

The site of residual cholesteatomas in closed techniques are, according to Aquino,^40^ 40.0% in the attic, 36.0% in the mesotympanic area, and 23.0% in other sites, confirming Wayoff et al's.^42^ results.

## CONCLUSION

Based on a survey of 1,146 cholesteatoma surgery cases we attempted to add knowledge about the epidemiology of this condition in the Brazilian context, given that published papers on this specific topic are rare.

We hope this study will be added to other Brazilian studies to understand the true situation of cholesteatomas in our country.
